# Role of stereotactic body radiation in the enhancement of the quality of life in locally advanced pancreatic adenocarcinoma: a systematic review

**DOI:** 10.1186/s13014-022-02076-5

**Published:** 2022-06-17

**Authors:** Marlies Vornhülz, Sofia Anton, Balint Eross, Zsolt Szakács, Peter Hegyi, Ivonne Regel, Claus Belka, Maximilian Niyazi, Julia Mayerle, Georg Beyer

**Affiliations:** 1 Department of Internal Medicine II, LMU University Hospital, Marchioninistr. 15, 81377 Munich, Germany; 2 Institute for Translational Medicine, Medical School, University of Pécs, Pécs, Hungary; 3 Department of Radiation Oncology, University Hospital, LMU, Munich, Germany; 4 Bavarian Center for Cancer Research (BZKF), Erlangen, Germany

**Keywords:** SBRT, Pancreatic ductal adenocarcinoma, Pancreatic cancer, PDAC, Radiotherapy, Palliative therapy, Symptom control, Quality of life, Qol, Pain, Biliary complications, Nutrition

## Abstract

**Introduction:**

Up to 30% of pancreatic cancer patients initially present locally advanced (LAPC). Stereotactic body radiation therapy (SBRT) may be an additional palliative treatment option when curative resection is no longer achievable. Our systematic review aimed to assess the effect of SBRT on the quality of life in LAPC.

**Methods:**

We searched five databases until June 29th, 2021, for original articles that reported on SBRT for histologically proven LAPC in adults. Data were extracted on study characteristics, SBRT and additional therapy regimen, pain, biliary complications, nutrition, quality of life and other patient-reported outcomes. Statistical analyses were performed for population and survival data.

**Results:**

11 case series studies comprising 292 patients with a median age of 66 (range 34–89) years were included in the final analysis. The weighted average BED2;10 (radiation biologically effective dose, equivalent dose in 2 Gy fractions) was 54 Gy, delivered in 3 to 6 fractions. The individual studies used different scales and endpoints, not allowing a meta-analysis. Pain generally appeared to be improved by SBRT. SBRT significantly reduced jaundice. Local control was achieved in 71.7% of patients. Weight loss and nausea also tended to improve after SBRT.

**Conclusion:**

SBRT of locally advanced irresectable pancreatic cancer is a promising approach for achieving local control and improving the quality of life. However, randomized controlled trials with larger cohorts are needed to assess the value of SBRT in pancreatic cancer therapy.

## Introduction

The incidence of pancreatic adenocarcinoma (PDAC) has been increasing in recent years [1] and is projected to be the second leading cause of cancer-related deaths in Europe and the U.S. within the next decade [2, 3]. Curative surgical resection is only possible in those 20–30% of small localised tumours without distant spreading [4]. The majority of cases is diagnosed when metastases are present; thus, curative treatment is no longer an option. Systemic chemotherapy remains the only approved palliative therapy [5]. In approximately a third of all cases, the tumour is diagnosed locally advanced, hence inoperable, yet still without metastases [6]. Patients with locally advanced pancreatic cancer (LAPC) usually receive neoadjuvant chemotherapy to achieve respectability through downstaging. However, due to insufficient response, resectability often cannot be reached. Furthermore, some patients cannot undergo surgery due to their impaired performance status. So far in these patients, only palliative chemotherapy has been shown to be effective in randomised controlled trials [7, 8], although the life expectancy of this group of non-resectable non-metastasized patients is longer than that of the metastasized group.

Many patients with LAPC suffer from pain, jaundice and weight loss [9]. Often, repetitive hospital stays and endoscopic interventions to relieve obstructive jaundice are needed. The patients' quality of life is henceforth gravely impaired. Since life expectancy is limited for PDAC patients, it is essential to offer the patients all options to improve quality of life. An improvement in the quality of life for PDAC patients should result in less pain, fewer hospital admissions and less medication to take.

Radiotherapy at the moment is not recommended as the first line treatment for pancreatic cancer therapy, but it can be used for symptom control and as part of tailored treatment approaches. Usually, it is applied in a combined chemoradiotherapy plan [10]. While chemotherapy helps to prolong overall survival for several months and inhibits the spread of metastases [11], it does not have a remarkable effect on the patients' symptoms. Yet, it has been shown that stereotactic body radiation therapy (SBRT) may improve the quality of life of LAPC patients [12].

SBRT is usually delivered in one to five fractions with a median cumulative dose of approx. 30 Gy, thus delivering high amounts of radiation in a relatively short time period with acceptable toxicity [13–15]. It may therefore be a chemo-sparring alternative palliative treatment, at relatively little expense and may be an option for frail patients with LAPC.

The impact of SBRT on the quality of life in LAPC is currently unknown. In this systematic review, we sought to answer whether SBRT reduces pain and biliary complications and improves nutritional status in locally advanced irresectable pancreatic adenocarcinoma.

## Methods

We report our systematic review and meta‐analysis in accordance with the Preferred Reporting Items for Systematic Reviews and Meta‐Analyses (PRISMA) Statement [16]. This systematic review was registered at the University of York international prospective register of systematic reviews (Prospero, CRD42019131081).

### Search strategy

A systematic search of MEDLINE (via PubMed), Cochrane Library, Embase, Scopus, Global Index Medicus, and Web of Science was performed until June 29th, 2021. We searched for all articles containing information on pancreatic cancer and SBRT. We decided to phrase the search term in order to detect all papers involving SBRT and pancreatic cancer since quality of life parameters are often only collected as secondary outcomes and thus restricting the search term to quality of life might have posed a risk of bias. The precise search term which was used in all databases is shown in the appendix. After eliminating duplicates, two independent investigators screened all records by title, abstract, and full-text (MV, SA). Discrepancies were resolved by discussion with a senior investigator (GB).

### Eligibility criteria

English or German language original articles reporting SBRT for LAPC in adults were included. Papers that included only metastatic disease, less than five patients or animal data where excluded. Reviews, letters and conference abstracts were excluded. Eligibility criteria are displayed in Table 1. Since there is no international consensus definition of SBRT, we have included all treatment strategies that were termed respectively by the authors in order not to miss any relevant reports.

**Table 1:** Eligibility criteria

Inclusion criteria	Exclusion criteria
Locally advanced pancreatic adenocarcinoma	Metastatic disease
English or German language	Any other language than English or German
Original article	Review, meta-analysis, letter to the editor, conference abstract or conference paper, case report or case series < 5 patients

### Data extraction, synthesis and analysis

Two authors (MV, SA) independently extracted data in a predesigned Excel 2016 sheet (Office 365, Microsoft, Redmond, WA, USA). Discrepancies were resolved by discussion with the senior investigator (GB). We extracted data on the study population, study type, cancer characteristics, SBRT regimen, additional therapy, survival data and most importantly, patient-reported outcomes (pain, biliary complications, nutrition, quality of life) and side effects of the treatment. Data synthesis was performed using the methods recommended by the working group of the Cochrane Collaboration [17]. Statistical analyses, where applicable, were performed using Excel. Weighted averages of the collected data were calculated. Ranges were combined from all provided ranges. The BED 2;10 was calculated from the data provided in the articles, where provided.

### Quality and risk of bias assessment

The quality of the studies was assessed by two independent examiners (MV, SA) using Oxford CEBM Levels 2011 [18].

## Results

### Search results

**Fig. 1:**
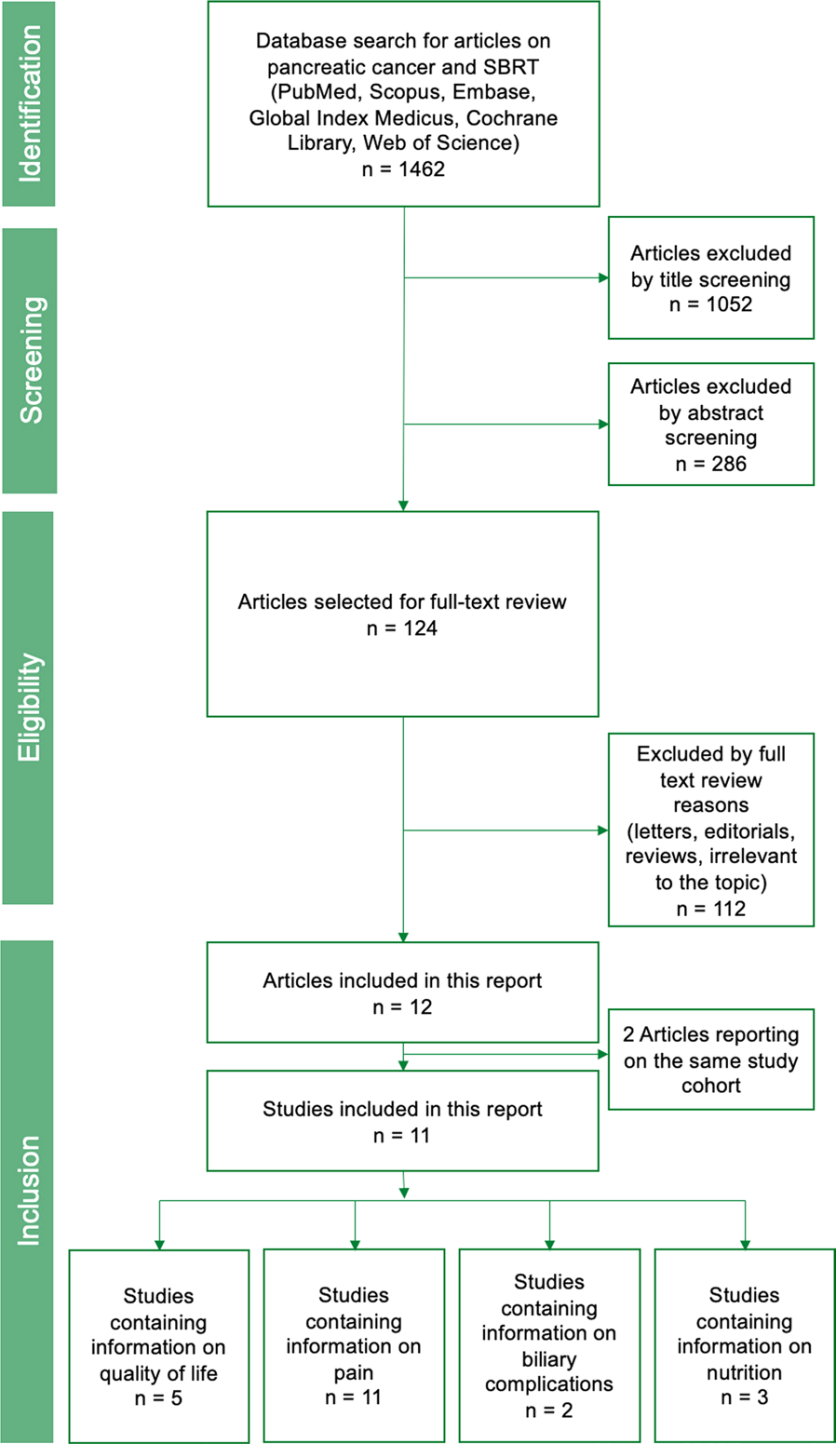
PRISMA flow chart of the screening and selection process

Our search terms identified 1.462 articles in the included databases. The search yield is shown in Fig. 1. After selection, 11 eligible studies were identified. Herman [19] and Rao [20] reported on the same study cohort of patients, which is why we synthesized the data and considered the study as one. All the studies included are displayed in Table 2 and listed in the appendix.

**Table 2:** Studies included in the final analysis

Author and Year	Location	Study type	Study period	Number of centers	Number of participants	Median age (years)	Gender ratio (m:f)	max. ECOG	Location of cancer (% head)	Previous CTx (%)	Previous RTx (%)	SBRT BED 2;10 (Gy)
Cozzi (2019)	Italy	retrospective data analysis	n/a	1	100	70.5	47:53	2	65.0	55	0	65.63
Gurka (2013)	USA	prospective clinical trial phase I	2009–2011	1	11	62.5	05:05	1	80.0	100	0	31.25
Herman (2015), Rao (2016)	USA	prospective clinical trial phase II	2010–2012	3	60	67	31:18	1	84.0	90	0	45.65
Hoyer (2005)	Denmark	prospective clinical trial phase II	2000–2001	2	22	61	10:12	2	n/a	0	0	93.75
Ji (2020)	China	retrospective data analysis	2017–2019	1	23	64	10:13	n/a	59.6	52	n/a	n/a
Jumeau (2018)	Canada	retrospective data analysis	2010–2016	1	21	69	07:13	n/a	52.0	38	0	37.50
Liauw (2020)	USA	prospective clinical trial phase I/II	2010–2016	1	15	61	06:09	1	n/a	100	n/a	76.95
Macchia (2012)	Italy	case series	n/a	1	16	61	11:05	2	56.0	94	56	32.6
Ryan (2018)	USA	retrospective data analysis	2010–2016	1	29	74	11:18	2	76.0	76	0	36.40
Shen (2010)	China	prospective observational trial	2009–2010	1	20	54	14:06	2	65.0	n/a	n/a	n/a
Tozzi (2013)	Italy	prospective clinical trial	2010–2011	1	30	67	20:10	2	70.0	100	0	62.69

*ECOG*, Performance Status assessed by Eastern Cooperative Oncology Group, *m* male, *f* female, *CTx* Chemotherapy, *RTx* radiotherapy, *BED2;10* radiation biologically effective dose, equivalent dose in 2 Gy fractions, *Gy* gray

All studies were either retrospective or prospective observational studies without control groups. Therefore, the quality of the studies was rated low (Level 4 on Oxford CEBM 2011).

### Population characteristics

The study periods covered a time frame from 1998 to 2019. Overall, 292 patients were included, with the study population size varying between 11 and 60 patients. The weighted average median age of observed patients was 66 (34–89) years. The gender ratio was balanced, slighty favouring male gender (1,2:1). Further population characteristics are displayed in Table 2. All patients suffered from pancreatic adenocarcinoma. In 70,9%, PDAC was located in the head of the pancreas. Cancer size (if documented) varied between 4 and 7.5 cm before SBRT. As predefined by our search terms, all studies' main focus was LAPC with Union for International Cancer Control (UICC) stage II-III. Nevertheless, one study included some patients with stage I cancer who were inoperable for medical reasons [21].

### Therapy regimens

SBRT regimens in the included studies varied. However, all of them met the characteristics needed to qualify for SBRT, that is a well-defined target volume with a small number of fractions and highly conformal dose distribution [15]. SBRT was performed employing different instruments, including CyberKnife, TrueBeam, Varian Trilogy and ClinArc, Siemens Primus and ClinAC 2100, as well as different techniques, including Rapidarc, and finally different modes of image-guidance, such as ConeBeam. The cumulative radiation dose administered ranged from 20 to 55 Gray. The therapy regimens varied between 3 and 6 fractions of 4 to 15 Gy/fraction. The weighted average BED2;10 was 54,21 Gy. 4% (range 0–100%) of patients received a form of previous radiotherapy, 73% (range 0–100%) received previous chemotherapy. The number of patients receiving concurrent chemotherapy in a chemoradiotherapy regimen varied greatly (0–100%). Concomitant chemotherapy data was not reported in all studies. The most common chemotherapy regimens were Gemcitabine and a combination of folinic acid, fluorouracil, irinotecan and oxaliplatin (FOLFIRINOX).

### Patient-reported outcomes

All eleven studies reported on any patient-reported outcomes [19–30]. Only five reported quality of life [19, 20, 23, 27–29]. The others detailed varying symptoms, including pain, biliary complications, nutritional status and nausea. The assessment protocols varied greatly, as did the time intervals of follow up. All of these studies had few participants (11–60 patients).

#### Quality of life

Quality of life was measured "directly" by questionnaires [19, 20, 23] in some studies; others measured surrogate parameters, such as performance status [27–29]. While quality of life of PDAC patients is usually severely impaired, the quality of life and the performance status taken as surrogate parameter appeared to be at the least stabilized by SBRT treatment.

Herman [19] and Rao [20] used the Quality of Life Questionnaire for cancer patients (QLQ-C30) and the Quality of Life Questionnaire for pancreatic cancer patients (QLQ-PAN26) questionnaire to assess changes in quality of life in their study population of 60 patients. With a baseline score of 67 (IQR 50–84), they did not observe significant changes in the global quality of life at 4–6 weeks or four months after SBRT. Gurka [23] also used the QLQ-C30 and the QLQ-PAN26 for quality of life assessment. With a small cohort, they obtained heterogeneous results, with 2/10 (20%) reporting an improvement in the quality of life, 2/10 (20%) reporting no change and 6/10 (60%) reporting a deterioration directly after SBRT. After one month, 2/8 (25%) reported an improvement, 2/8 (25%) no change and 4/8 (50%) deterioration.

Macchia [27] assessed the performance status by the Eastern Cooperative Oncology Group (ECOG) scale. Here, they saw stable or improved values in 75% of their 16 patients four weeks after SBRT. Furthermore, the cancer linear analogue scale (CLAS) was used to assess the quality of life (CLAS1), level of energy (CLAS2) and the ability to undertake daily activities (CLAS3). CLAS1 and CLAS3 were stable or improved in 75% of patients, while CLAS2 was stable or improved in 56% after treatment. Ryan [28] also measured the performance status on the ECOG scale; 3/13 (23%) patients with an initial ECOG of 2 improved to 1 or 0, while 1/16 (6%) patient with ECOG 0 or 1 declined to 2. Shen [29] used the Karnofsky score to assess the performance status; it increased in 15/20 (75%), did not change in 3/20 (15%) and decreased in 2/20 (10%) patients 3 months after SBRT.

#### Pain

Concerning pain as one of the main complaints in patients with LAPC, all eleven studies comprising 292 patients reported on it in significantly more detail. Generally, pain appears to be improved by SBRT therapy [19–30]. It also appeared that the maximum pain palliation effect was achieved at approx. one month after SBRT while pain levels were increasing again afterwards. An overview is displayed in Table 3.

**Table 3:** Assessment of pain

Publication	Assessment tool	Time after SBRT (Change of pain in % of patients compared to the number of patients reporting pain at baseline)			
Comito (2017)	NRS	*1 month* improvement in 82% (62% analgesics suspended, 10% analgesics reduced by 50%, 10% analgesics reduced by 20%)		*3 months* improvement in 82% (62% analgesics suspended, 10% analgesics reduced by 50%, 10% analgesics reduced by 20%)	
Gurka (2013)	QLQ-PAN26	*directly after radiotherapy* improvement 50%, no change 20%, worsening 30%		*1 month* improvement 63%, no change 12%, worsening 25%	
Herman (2015), Rao (2016)	QLQ-PAN26	*4–6 weeks* improvement 100%		*4 months* return to baseline 100%	
Hoyer (2005)	CTCAE	*Baseline* 54% pain ≥ 2	*2 weeks* 71% pain ≥ 2	*3 months* 94% pain ≥ 2 80% on morphine	*Later on* some transient improvement
Ji (2020)	NRS	*Baseline* 4.6 ± 1.3	*4 weeks* 2.5 ± 2.1	*2 months*3.0 ± 2.4	*3 months*3.1 ± 2.4
Jumeau (2018)	CTCAE	*1 month* improvement 36%, worsening 14% (of initially pain-free)			
Liauw (2020)	NRS	8/15 patients had pain before SBRT, 5/8 (63%) had pain response after SBRT			
Macchia (2012)	VAS	*4 weeks* improvement 44% (reduction of pain medication 22%)			
Ryan (2018)	unknown	*3 months* improvement 73%			
Shen (2010)	VAS	*3 months* improvement 100%			
Tozzi (2013)	NRS	*1 month* improvement 100% (64% analgesics suspended, 27% analgesics reduced by 50%, 9% analgesics reduced by 20%), worsening 10% (of initially pain-free)			

*CTCAE* common terminology criteria for adverse events, *NRS* numerical rating scale for pain, *QLQ-C30* quality of life questionnaire for cancer patients, *QLQ-PAN26* quality of life questionnaire for pancreatic cancer patients, *VAS* visual analog scale for pain

Herman [19] and Rao [20] again by QLQ-PAN26 questionnaire measured a significant decrease of pain in 100% of patients at the first follow up at 4–6 weeks after SBRT. However, they saw a return to baseline at the second follow up at 4 months. Gurka [23], quantifying pain also by QLQ-PAN26, found an improvement in 50%, a stable level of pain in 20% and worsening pain in 30% of their patients directly after SBRT. One month after therapy, 63% reported an improvement in pain, 12% no change and 25% worsening pain.

Comito [22], Ji [24], Liauw [26] and Tozzi [30] evaluated pain using the Numerical Rating Scale (NRS) scoring system. According to Comito [22], 39% of their 45 patients experienced pain before SBRT according to the Numerical Rating Scale (NRS) scoring system. In follow-ups one month and three months after SBRT, pain improved in 62% to a level where analgesic administration could be suspended; in 10%, analgesics could be reduced by 50%, and in 10%, administration was reduced by 20%. In the study by Tozzi [30], 37% of the 30 patients suffered from pain before SBRT. Complete pain control was achieved in 64% so that analgesics administration could be suspended. In 27% of patients, analgesics dosage could be reduced by 50%. In 9% of patients, analgesics dosage could be reduced by 20% one month after SBRT. 10% of initially pain-free patients developed grade 2 (CTCAE) pain. In the study by Liauw [26], 53% of the 15 patients suffered from pain before SBRT with a median NRS rating of 2. 63% of these patients showed an improvement of pain after SBRT. Contrastingly to the other studies, Ji [24] reported that all patients suffered pain at baseline. They reported an average NRS rating of 4.6 on NRS at baseline, with the NRS on average decreasing to a minimum NRS of 2.5 four weeks after SBRT and eventually slightly increasing again up until 3.1 at three months after SBRT, yet never reaching baseline levels again in the follow-up time. It should be stressed that in this group oral morphin consumption increased over time compared to baseline, although not statistically significant.

Hoyer [21], as well as Jumeau [25], graded pain on a toxicity scale from 0 to 4 according to CTCAE. In the study by Hoyer [21], 54% of patients reported a pain level pain ≥ 2 at baseline, two weeks after treatment, 71% of patients reported a pain level ≥ 2. At three months, 94% of patients had pain ≥ 2, and 80% received morphine; however, some patients reported a transient improvement later on. In the study by Jumeau [25], 52% of the 21 patients reported pain at baseline. At follow-up one month after SBRT, 36% reported a decrease in pain. Among the initially asymptomatic patients, two developed grade 2 pain (according to CTCAE) and one developed grade 3 pain due to local progression.

In the study by Macchia [27], 44% of the 9 patients who initially reported pain showed a complete or partial pain relief four weeks after SBRT calculated by VAS and pain score. 22% could reduce their intake of pain medication. Ryan [28] reported symptom palliation in 73% of the 11 patients who initially reported pain at the 3 months follow up. In the study by Shen [29], a 90% pain relief rate in the 15 patients who reported pain by the visual scoring method was achieved 3 months after treatment.

#### Biliary complications

While biliary complications are common in PDAC patients and are known to pose an impact on quality of life in PDAC patients [31], only two studies reported these [19, 20, 29]. A summary is displayed in Table 4. QLQ-PAN26 jaundice scores were significantly improved in the study by Herman [19] and Rao [20] after 4–6 weeks and four months, but without correlating clinical significance. In the 13 out of 20 patients who initially had relevant jaundice, Shen [29] could show a remission rate of 77%. Before treatment, the mean values of total bilirubin and combined bilirubin were 114 µmol/L (45–354 µmol/L) and 24 µmol/L (8–103 µmol/L). After treatment, the mean values were 15 µmol/L (3–42 µmol/L) and 4 µmol/L (2–14 µmol/L), respectively. Herman [19] and Rao [20] admitted though that the impact of biliary stenting was not examined. Shen [29] provided no details on endoscopic interventions either. No data on pruritus, cholangitis or cholangiosepsis were provided. Since it may be assumed that biliary obstruction caused by a pancreatic head tumour may be improved when local control is achieved, local control should also be highlighted. One-year local control was achieved in 71,7% of patients after SBRT.

**Table 4:** Assessment of biliary complications

Publication	Time points	Assessment tool	Effect
Herman (2015), Rao (2016)	4–6 weeks and four months	QLQ-PAN26 jaundice scores	Significant improvement
Shen (2010)	Before and after SBRT	Total bilirubin (mean, range)	114 µmol/L (45­354 µmol/L) reduced to 15 µmol/L (3­42 µmol/L)
		Combined bilirubin (mean range)	24 µmol/L (8­103 µmol/L) reduced to 4 µmol/L (2­14 µmol/L)

*SBRT* stereotactic body radiation therapy, *QLQ-PAN26* quality of life questionnaire for pancreatic cancer patients

#### Nutritional status and nausea

Herman [19] and Rao [20] did not directly report on changes in body weight or the symptom of nausea; however, they reported on the body image dissatisfaction evaluated by the QLQ-PAN26 questionnaire. Here, they could eventually report a significant decrease in body image dissatisfaction after four months, when there had initially been no change after 4–6 weeks. Macchia [27] could not detect a significant change in the regularly measured body weight after treatment. Ryan [28] described symptom palliation after three months in 58% of their patients suffering from anorexia, 80% for weight loss, and 100% for nausea. Hoyer [21] reported nausea similar to pain graded according to CTCAE. Initially, 9% of patients reported nausea ≥ 2. After 14 days, they observed an increase of 100% in nausea ≥ 2, whereas, after three months, 25% of patients experienced less nausea compared to baseline.

## Discussion

With the recent advances in overall survival accomplished by dual chemotherapy in advanced pancreatic adenocarcinoma, the enhancement of those patients’ quality of life rises into focus. Equivalently, genuine therapeutic options are needed for those patients who are medically unfit for chemotherapy or choose not to undergo any. SBRT may provide symptom control, thus resulting in a better quality of life of affected patients. In our systematic review, we found several original articles focusing on the effect of SBRT on the quality of life in LAPC. As of today, there is no published randomized controlled trial on the role of SBRT to control the symptoms of LAPC. Moreover, the published articles only reported on retrospective case series with small cohorts devoid of control groups. Chemotherapy, which must be considered an important factor for the quality of life of affected patients, was not sufficiently recorded by all studies, hence not allowing to calculate or specifically discuss any differences. This systematic review thus provides a qualitative rather than quantitative analysis on SBRT for quality of life in patients with metastasized PDAC.

Previous studies have already shown that SBRT is beneficial for pain in PDAC [12, 32]. Our analysis confirmed these results showing that SBRT treatment reliably reduces pain in PDAC patients. However, the included studies were very inconsistent in reporting on symptoms or medication use before and after therapy. Moreover, assessment scales varied considerably, hence a quantitative meta-analysis was not possible. Nonetheless is appears that it was possible to reduce pain medication or even discontinue it altogether in a significant number of patients.

Biliary complications, that is, jaundice or cholangitis due to biliary obstruction, also play an essential role in PDAC patients' QoL. Their relevance is reflected in their prominent position in the validated Functional Assessment of Cancer Therapy Hepatobiliary Symptom Index (FHSI-8) [31], which is commonly used to assess quality of life in patients suffering from cancers of hepatobiliary or pancreatic origin. Biliary complications often lead to hospital stays, interventions, antibiotic treatment and possibly a delay of tumour-specific therapy. Especially tumours of the pancreatic head often cause biliary obstruction, which is why their local control, e.g. by SBRT, can be conjectured to be beneficial in this respective. Unfortunately, only two study groups examined the number of biliary complications. In both studies, jaundice significantly improved after SBRT. However, they did not comment on the clinical significance nor reported on biliary stenting, which introduces a bias and does not allow us to further elucidate this topic. Nonetheless, it may be assumed that jaundice caused by a pancreatic head tumour is reduced when local control is achieved. 1-year local control was achieved in more than 70% of patients, hence appearing rather successful. It would even be interesting to assess the cost-effectiveness of SBRT by reducing biliary complications, with biliary obstruction being one of the leading causes of hospitalization in the affected patients.

Weight loss is a common symptom of PDAC patients. Related to this, pain, nausea, and appetite loss are reported as underlying causes in these patients. While this may severely reduce the quality of life of PDAC patients, almost no study reported data on this symptom group. There appears to be a tendency towards improving these symptoms; however, more reliable and more standardized data are warranted.

Many of the patient-reported outcomes were assessed by different scales and/or questionnaires in the included studies. QoL was measured directly by questionnaires in three studies only [19, 20, 23]. However, no significant changes in quality of life were shown, but deterioration was reported for some patients in some studies. One could argue, though, that QoL will deteriorate with the disease's progression, and thus deterioration was not due to SBRT. Some other studies measured the patients' performance status, which may be considered a surrogate marker for the QoL. Here, a tendency towards increased performance status could be observed.

Quality of life is always the bigger picture comprised of many facets contributing to it. For patients suffering from pancreatic carcinoma, the experience of pain greatly impairs quality of life. Moreover, other factors such as nutrition, biliary compications, mobility and hospital stays influence the QoL [31, 33]. While only very few studies explicitly examined quality of life, those underlying factors have been investigated. Most importantly, SBRT appears to provide reasonable pain control. At the same time, there are indications that it may also improve other symptoms such as biliary complications and nutritional status, thereby improving the QoL of PDAC patients, even though not prolonging life. At the same time, SBRT does not cause grossly increased toxicity. Thus, SBRT may be a good option for inoperable patients due to locally advanced cancer or poor functional performance status [34]. Apart from the small number of studies presenting patient-reported outcomes, the existing studies are rarely more than retrospective in-house analyses, and randomized-controlled trials are missing. The cohorts reported are relatively small; often, they comprise less than twenty patients, the patient cohorts themselves being rather inhomogenous. Since the data result from in-house analyses from different years, the therapeutic regimens vary substantially. All in all, this reduces the significance of the results considerably. As in previous systematic reviews [32], a quantitative meta-analysis was not possible. Yet, qualitative evaluation showed promising options to improve the QoL of patients employing SBRT in addition to systemic therapy.

In conclusion, stereotactic body radiation therapy of locally advanced pancreatic cancer appears to provide a promising approach to improve the quality of life of affected patients. However, studies with larger cohorts and higher quality are needed to assess the value in pancreatic cancer therapy.

## Data Availability

Data extraction sheet submitted, all other information available on request.

## Appendix

A. Search Term
  ((((pancreas OR pancreatic) AND (cancer OR carcinoma OR adenocarcinoma OR malignan* OR tumour OR tumor)) OR PDAC)) AND (SBRT OR (stereotactic AND (radiation OR radiotherapy))).
B. List of all included articles
  Comito, T., et al. (2017). "Can Stereotactic Body Radiation Therapy Be a Viable and Efficient Therapeutic Option for Unresectable Locally Advanced Pancreatic Adenocarcinoma? Results of a Phase 2 Study." Technology in Cancer Research and Treatment **16**(3): 295–301.
  Gurka, M. K., et al. (2013). "Stereotactic body radiation therapy with concurrent full-dose gemcitabine for locally advanced pancreatic cancer: A pilot trial demonstrating safety." Radiation Oncology **8**(1).
  Herman, J. M., et al. (2015). "Phase 2 multi-institutional trial evaluating gemcitabine and stereotactic body radiotherapy for patients with locally advanced unresectable pancreatic adenocarcinoma." Cancer **121**(7): 1128–1137.
  Hoyer, M., et al. (2005). "Phase-II study on stereotactic radiotherapy of locally advanced pancreatic carcinoma." Radiotherapy and Oncology **76**(1): 48–53.
  Ji, K., et al. (2020). "Celiac Plexus Block After Stereotactic Body Radiotherapy Improves Pain Relief in Locally Advanced Pancreatic Cancer." J Pain Res **13**: 919–925.
  Jumeau, R., et al. (2018). "Stereotactic body radiotherapy (SBRT) for patients with locally advanced pancreatic cancer: A single center experience." Digestive and Liver Disease **50**(4): 396–400.
  Liauw, S. L., et al. (2020). "A prospective trial of stereotactic body radiation therapy for unresectable pancreatic cancer testing ablative doses." J Gastrointest Oncol **11**(6): 1399–1407.
  Macchia, G., et al. (2012). "Quality of life and toxicity of stereotactic radiotherapy in pancreatic tumors: A case series." Cancer Investigation **30**(2): 149–155.
  Rao, A. D., et al. (2016). "Patient-reported outcomes of a multicenter phase 2 study investigating gemcitabine and stereotactic body radiation therapy in locally advanced pancreatic cancer." Practical Radiation Oncology **6**(6): 417–424.
  Ryan, J. F., et al. (2018). "Stereotactic body radiation therapy for palliative management of pancreatic adenocarcinoma in elderly and medically inoperable patients." Oncotarget **9**(23): 16,427–16,436.
  Shen, Z. T., et al. (2010). "Preliminary efficacy of cyberknife radiosurgery for locally advanced pancreatic cancer." Chinese Journal of Cancer **29**(9): 802–809.
  Tozzi, A., et al. (2013). "SBRT in unresectable advanced pancreatic cancer: Preliminary results of a mono-institutional experience." Radiation Oncology **8**(1).
C. Prisma Checklist

## Abbreviations

BED2;10: Radiation biologically effective dose, equivalent dose in 2 Gy fractions
CEBM: Centre for Evidence-Based Medicine
CLAS: Cancer linear analogue scale
CTCAE: Common Terminology Criteria for Adverse Events
CTx: Chemotherapy
ECOG: Eastern Cooperative Oncology Group
FOLFIRINOX: Chemotherapy regimen comprising folinic acid, fluorouracil, irinotecan and oxaliplatin
Gy: Gray
LAPC: Locally advanced pancreatic carcinoma
NRS: Numerical Rating Scale for Pain
PDAC: Pancreatic ductal adenocarcinoma
RTx: Radiotherapy
SBRT: Stereotactic body radiation therapy
UICC: Union for International Cancer Control
USA: United States of America
QLQ-C30: Quality of Life Questionnaire for cancer patients
QLQ-PAN26: Quality of Life Questionnaire for pancreatic cancer patients
QoL: Quality of Life
VMAT: Volumetric modulated arc therapy
VAS: Visual Analog Scale for Pain
